# Supplementary material for: Cholinergic blockade reveals a role for human hippocampal theta in memory encoding but not retrieval
Source: eLife. 2026 Nov 11;14:RP108972. doi: 10.7554/eLife.108972 (PMC13754277; doi:10.7554/eLife.108972)
Supplement: Supplementary file 2 [file elife-108972-supp1.pdf]

Supplementary File

Subject ID	Gender	AH	PH	EC
S1	M	2	3	0
S2	M	4	3	1
S3	M	3	3	2
S4	M	4	4	2
S5	M	5	3	1
S6	M	5	4	1
S7	F	4	2	4
S8	M	2	2	0
S9	F	3	1	2
S10	F	6	0	0
S11	F	4	4	3
S12	M	4	3	0

Supplementary File 1. Subject demographics and electrode coverage. Table showing subject demographics and number of electrodes across all regions of interest, including the anterior hippocampus (AH), the posterior hippocampus (PC), and the entorhinal cortex (EC).
