# Supplementary material for: Cholinergic blockade reveals a role for human hippocampal theta in memory encoding but not retrieval
Source: eLife. 2026 Nov 11;14:RP108972. doi: 10.7554/eLife.108972 (PMC13754277; doi:10.7554/eLife.108972)
Supplement: Peer review history

# Editor report: eLife Assessment

**DOI:** 10.7554/eLife.108972.3.sa0

**Authors:** Gui Xue

**Affiliations:** Beijing Normal University China

**Keywords:** Convincing, Fundamental

This **fundamental** work advances our understanding of the role of human hippocampal theta oscillations in memory encoding and retrieval. The evidence supporting the conclusions is **convincing**, using both scopolamine administration and intracranial EEG recordings. This work will be of broad interest to neuroscientists and has translational implications.

# Referee report: Reviewer #1 (Public review)

**DOI:** 10.7554/eLife.108972.3.sa1

Summary:

The authors report intracranial EEG findings from 12 epilepsy patients performing an associative recognition memory task under the influence of scopolamine. They show that scopolamine administered before encoding disrupts hippocampal theta phenomena and reduces memory performance, and that scopolamine administered after encoding but before retrieval impairs hippocampal theta phenomena (theta power, theta phase reset) and neural reinstatement but does not impair memory performance. This is an important study with exciting, novel results and translational implications. The manuscript is well written, the analyses are thorough and comprehensive, and the results seem robust.

Strengths:

- Very rare experimental design (intracranial neural recordings in humans coupled with pharmacological intervention);

- Extensive analysis of different theta phenomena;

- Well-established task with different conditions for familarity versus recollection;

- Clear presentation of findings;

- Translational implications for diseases with cholinergic dysfuction (e.g., AD);

- Findings challenge existing memory models and the discussion presents interesting novel ideas.

# Referee report: Reviewer #2 (Public review)

**DOI:** 10.7554/eLife.108972.3.sa2

Summary:

In this study, performed in human patients, the authors aimed at dissecting out the role of cholinergic modulation in different types of memory (recollection-based vs familiarity and novelty-based) and during different memory phases (encoding and retrieval). Moreover, their goal was to obtain the electrophysiological signature of cholinergic modulation on network activity of the hippocampus and the entorhinal cortex.

Strengths:

Authors combined cognitive tasks and intracranial EEG recordings in neurosurgical epilepsy patients. The study confirms previous evidence regarding the deleterious effects of scopolamine, a muscarinic acetylcholine receptor antagonist, on memory performance when administered prior the encoding phase of the task. During both encoding and retrieval phases scopolamine disrupts the power of theta oscillations in terms of amplitude and phase synchronization. These results raise the question on the role of theta oscillations during retrieval and the meaning of scopolamine effect on retrieval-associated theta rhythm without cognitive changes. The authors clearly discussed this issue in the discussion session.

A major point is the finding that scopolamine-mediated effect is selective for recollection-based memory and not for familiarity- and novelty-based memory.

The methodology used is powerful and the data underwent a detailed and rigorous analysis.

# Author comment: Author response

**DOI:** 10.7554/eLife.108972.3.sa3

**Authors:** Tamara Gedankien, Jennifer Kriegel, Erfan Zabeh, David McDonagh, Bradley C Lega, Joshua Jacobs

**Affiliations:** Columbia University New York United States; The University of Texas Southwestern Medical Center Dallas United States; Department of Biomedical Engineering, Columbia University, New York, NY, USA New York United States; The University of Texas Southwestern Medical Center Dallas United States; University of Texas - Southwestern Medical Center Dallas United States; Columbia University New York United States

The following is the authors’ response to the original reviews.

**Public Reviews:**

**Reviewer #1 (Public review):**

Summary:

The authors report intracranial EEG findings from 12 epilepsy patients performing an associative recognition memory task under the influence of scopolamine. They show that scopolamine administered before encoding disrupts hippocampal theta phenomena and reduces memory performance, and that scopolamine administered after encoding but before retrieval impairs hippocampal theta phenomena (theta power, theta phase reset) and neural reinstatement but does not impair memory performance. This is an important study with exciting, novel results and translational implications. The manuscript is well-written, the analyses are thorough and comprehensive, and the results seem robust.

Strengths:

(1) Very rare experimental design (intracranial neural recordings in humans coupled with pharmacological intervention).

(2) Extensive analysis of different theta phenomena.

(3) Well-established task with different conditions for familiarity versus recollection.

(4) Clear presentation of findings and excellent figures.

(5) Translational implications for diseases with cholinergic dysfunction (e.g., AD).

(6) Findings challenge existing memory models, and the discussion presents interesting novel ideas.

Weaknesses:

(1) One of the most important results is the lack of memory impairment when scopolamine is administered after encoding but before retrieval (scopolamine block 2). The effect goes in the same direction as for scopolamine during encoding (p = 0.15). Could it be that this null effect is simply due to reduced statistical power (12 subjects with only one block per subject, while there are two blocks per subject for the condition with scopolamine during encoding), which may become significant with more patients? Is there actually an interaction effect indicating that memory impairment is significantly stronger when scopolamine is applied before encoding (Figure 1d)? Similar questions apply to familiarity versus recollection (lines 78-80). This is a very critical point that could alter major conclusions from this study, so more discussion/analysis of these aspects is needed. If there are no interaction effects, then the statements in lines 84-86 (and elsewhere) should be toned down.

The reviewer highlights important concerns regarding the statistical power of the behavioral effects. We address these concerns in the revised manuscript in two ways: (1) we provide a supplemental analysis using a matched number of blocks between the placebo and scopolamine conditions to avoid statistical bias related to differing trial counts, and (2) we include a supplemental figure illustrating paired comparisons between blocks.

(2) Further, could it simply be that scopolamine hadn't reached its major impact during retrieval after administration in block 2? Figure 2e speaks in favor of this possibility. I believe this is a critical limitation of the experimental design that should be discussed.

The reviewer raises an important methodological concern regarding the time required for scopolamine's effect to manifest and the subsequent impact on the study outcomes. Previous studies report that the average time to maximum serum concentration after intravenous (IV) scopolamine administration is approximately 5 minutes (Renner et al., 2005), with the corresponding clinical onset estimated at 10 minutes. In our study, the retrieval period in Block 2 commenced at 15 ± 0.2 post-injection across all subjects. Given this timing, there is sufficient reason to conclude that scopolamine had reached its major impact during the Block 2 retrieval phase. Furthermore, the observation of significant disruptions to theta oscillations during this same retrieval phase provides strong evidence that the drug was in full effect at that time.

(3) It is not totally clear to me why slow theta was excluded from the reinstatement analysis. For example, despite an overall reduction in theta power, relative patterns may have been retained between encoding and recall. What are the results when using 1-128 Hz as input frequencies?

Slow theta (2–4 Hz) was excluded from the reinstatement analysis to avoid potential confounding effects. Given the observed disruption to slow theta power following scopolamine administration, any subsequent changes in slow theta reinstatement would be causally ambiguous, potentially arising directly from the power effects. Therefore, we would be unable to determine whether changes in slow theta reinstatement were genuinely independent of changes in power.

(4) In what way are the results affected by epileptic artifacts occurring during the task (in particular, IEDs)?

To exclude abnormal events and interictal activity, a kurtosis threshold of 4 was applied to each trial, effectively filtering out segments exhibiting significant epileptic artifacts.

**Reviewer #2 (Public review):**

Summary:

In this study, performed in human patients, the authors aimed at dissecting out the role of cholinergic modulation in different types of memory (recollection-based vs familiarity and novelty-based) and during different memory phases (encoding and retrieval). Moreover, their goal was to obtain the electrophysiological signature of cholinergic modulation on network activity of the hippocampus and the entorhinal cortex.

Strengths:

The authors combined cognitive tasks and intracranial EEG recordings in neurosurgical epilepsy patients. The study confirms previous evidence regarding the deleterious effects of scopolamine, a muscarinic acetylcholine receptor antagonist, on memory performance when administered prior to the encoding phase of the task. During both encoding and retrieval phases, scopolamine disrupts the power of theta oscillations in terms of amplitude and phase synchronization. These results raise the question of the role of theta oscillations during retrieval and the meaning of scopolamine's effect on retrieval-associated theta rhythm without cognitive changes. The authors clearly discussed this issue in the discussion session. A major point is the finding that the scopolamine-mediated effect is selective for recollection-based memory and not for familiarity- and novelty-based memory.

The methodology used is powerful, and the data underwent a detailed and rigorous analysis.

Weaknesses:

A limited cohort of patients; the age of the patients is not specified in the table.

To comply with human subject privacy protection policies, age was not reported; however, we did not find any significant effects of age on the behavioral or neural measures.

**Recommendations for the authors:**

**Reviewer #1 (Recommendations for the authors):**

(1) Regarding dosage, did you take the patients' body weight into account? Do the effects hold when controlling for it?

We controlled for participant weight, yet the observed effects were more strongly correlated with the absolute scopolamine dosage, irrespective of weight. This outcome indicates that scopolamine likely rapidly crosses the blood-brain barrier, producing swift effects that are not initially influenced by metabolic variability.

(2) Line 96: Corrected for what kind of multiple comparisons?

We apologize for this confusion. The statistical analysis presented in this line does not require multiple-comparison correction, and we will therefore remove the annotation.

(3) Line 165: These are very interesting results. How do they relate to Rizzuto et al., NeuroImage, 2006?

Our findings show that successful retrieval is tied to an encoding-retrieval phase match, which is a refinement and application of the Rizzuto et al. (2006) work. Rizzuto et al. showed that memory events are phase-locked; we show that maintaining a specific, matched phase relationship between encoding and retrieval events is critical for memory success, and that this process is dependent on the cholinergic system.

**Reviewer #2 (Recommendations for the authors):**

Figure 1b: It would be useful for clarity to have the cartoon of the treatment paradigm for the encoding phase (blocks 3 and 4).

The treatment paradigm only involved a single intravenous (IV) injection of scopolamine (or saline, for the placebo condition). The injections were administered by the participant's attending nurse, with a board-certified anesthesiologist present at the time of injection and available throughout the experiment. These details are fully documented in the Methods section.
